# Soft Pneumatic Gripper With a Tendon-Driven Soft Origami Pump

**DOI:** 10.3389/fbioe.2020.00461

**Published:** 2020-05-13

**Authors:** Yeunhee Kim, Youngsu Cha

**Affiliations:** Center for Intelligent & Interactive Robotics, Korea Institute of Science and Technology, Seoul, South Korea

**Keywords:** biomimetics, origami, soft gripper, soft robotics, soft pump

## Abstract

In this study, we propose a soft pneumatic gripper that uses a tendon-driven soft origami pump. The gripper consists of three pneumatic soft actuators that are controlled by a tendon-driven origami pump. An external air compressor that supplies air to the pneumatic actuator is replaced by an origami pump. The soft actuator is composed of silicone (Ecoflex 00-30) with a chamber-based structure, which is fabricated using a mold, and the origami pump is fabricated by folding a Kresling patterned polypropylene film. In addition, we conduct a series of experiments to evaluate the performance of the pneumatic actuator with a tendon-driven origami pump. Specifically, movement characteristics, frequency response, blocking force, and the relation between bending angle and pressure are analyzed from the results of the experiments. Furthermore, we understand the entire operation mechanism from the deformation of the origami pump to bending through pressure. Finally, we demonstrate the grasping of objects with diverse shapes and materials, and indicate the feasibility of the pneumatic gripper as an independent module without an external compressor.

## 1. Introduction

Soft robotics has recently received considerable attention in the field of robotics. Soft robots, such as locomotive robots (Chan et al., 2012; Tolley et al., 2014; Wang et al., 2014), biomimetic robots (Laschi et al., 2009; Marchese et al., 2014; Snell-Rood, 2016; Lu et al., 2017; Della Santina et al., 2019), origami robots (Onal et al., 2012; Paez et al., 2016; Salerno et al., 2016; Firouzeh and Paik, 2017; Li et al., 2017; Lee and Rodrigue, 2019), micro robots (Li et al., 2016; Palagi et al., 2016), and soft manipulator, utilize the flexibility of soft materials (Hughes et al., 2016) in various applications.

The precious concept source for soft robotics corresponds to biomimetics. It mimics the movement and morphological characteristics of animals and plants (Laschi et al., 2009; Marchese et al., 2014; Wang et al., 2014; Snell-Rood, 2016; Lu et al., 2017; Della Santina et al., 2019). It is also inspired by motions and structures of the human body (Lu et al., 2017; Della Santina et al., 2019). A Human-like robotic manipulator is an extremely important and necessary research area. Various robotic manipulators are developed due to the explosive demand for robotic hands in manufacturing. They can perform various tasks in several unpredictable situations (Hughes et al., 2016). In contrast to conventional manipulators with a rigid body (Butterfaß et al., 2001; Wojtara et al., 2005), soft manipulators are actuated by various methods, such as hydraulic (Mitchell et al., 2019; Park et al., 2020) and pneumatic (Deimel and Brock, 2016; Jittungboonya and Maneewarn, 2019), and smart materials, such as ionic polymer metal composite (IPMC) (Carrico and Leang, 2017; Bhattacharya et al., 2019; Roy et al., 2019), shape memory alloy (SMA) (Rodrigue et al., 2017; Lee et al., 2019), and electroactive polymer (Kofod et al., 2007; Taghavi et al., 2018).

In this study, we propose a soft pneumatic gripper with a novel tendon-driven soft origami pump. Specifically, we utilize an origami pump as an air supplier for the pneumatic gripper instead of typical air compressors. Origami structures are adapted in various fields such as soft robots (Onal et al., 2012), actuators (Firouzeh and Paik, 2017), and artificial muscles (Li et al., 2017; Lee and Rodrigue, 2019) with various materials, such as paper (Paez et al., 2016), film (Onal et al., 2012; Li et al., 2017), and silicone (Sun et al., 2013). Origami can easily be patterned and folded in 2D planes to make 3D structures (Demaine and O'Rourke, 2007; Lang, 2011). We design an origami pump with a Kresling pattern, which can be folded in the form of a 3D cylinder shape from 2D patterned triangles (Kobayashi et al., 1998; Mahadevan and Rica, 2005; Kresling, 2008; Jianguo et al., 2015). The benefits of the origami pump include fast reaction speed, light weight, and reliable features. Also, the origami pump can facilitate modular pneumatic actuator through independent configuration.

To control the origami pump, we select the tendon-driven method. The tendon-driven mechanism is a commonly used method for conventional robot hands (Hong et al., 2018) and soft grippers (Lee et al., 2019). In our case, the tendon is connected between the origami pump and a motor. We control the cylinder height of the origami pump via the tendon. The tendon controls the origami pump with a sealed air and it can make the pump to have a small operating range than typical pumps. In addition, we experimentally and theoretically analyze the actuation performance of a finger component in the pneumatic gripper. Specifically, we examine the movement characteristics, frequency response, blocking force, and the relation between pressure and bending angle. Additionally, we demonstrate the grasping performance of the soft gripper with diverse objects.

This paper is organized as follows: In section 2, we describe the design of the soft finger module with a soft origami pump, including the fabrication method, operation principle, and experimental setup. In section 3, we show and analyze experimental results of the soft pneumatic finger. Specifically, the relations between input from the origami pump, pressure, and performance of pneumatic actuator are investigated and compared with that of the theoretical model. Additionally, the frequency response of the soft pneumatic actuator is analyzed in the section. In addition, we describe the design of the soft gripper consisting of three finger modules, and demonstrate the grasping performance with various objects. The conclusions are summarized in section 4.

## 2. Materials and Methods

### 2.1. Fabrication Method

**Figure 1:**
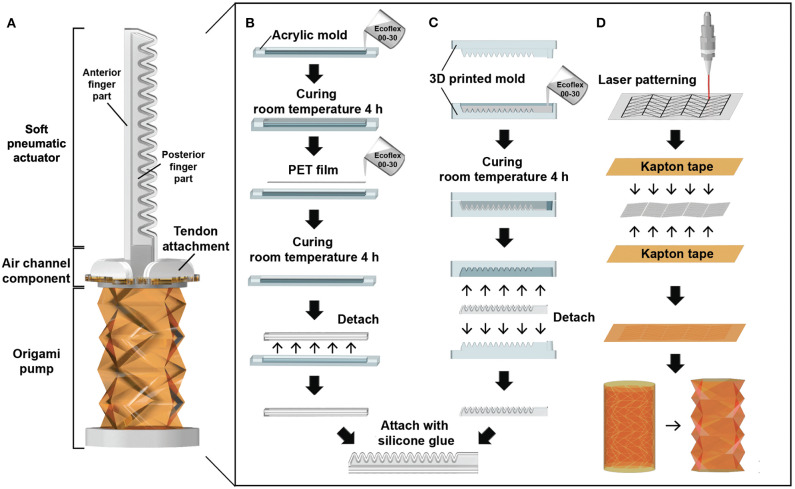
Fabrication process of a soft pneumatic actuator and an origami pump: **(A)** Soft finger module. **(B)** Fabrication process of the anterior finger part. **(C)** Fabrication process of the posterior finger part. **(D)** Fabrication process of the origami pump.

The soft finger module consists of a soft pneumatic actuator, air channel component, and origami pump (Figure 1A). Specifically, the soft pneumatic actuator is composed of two components: the anterior finger part and posterior finger part. Figure 1B presents the fabrication process of the anterior finger part. The mold is a combination of two acrylic bodies manufactured by a laser cutter (Epilog laser MINI 18, Epilog laser Corp.). The first step involves pouring the silicone solution (Ecoflex 00-30, Smooth-On, Inc.) in 1/3 of the mold and curing at room temperature for 4 h. Subsequently, a polyethylene terephthalate (PET) film with a thickness of 100 μm is attached to the cured silicone. The PET film is inserted as a backbone to increase the stiffness of the actuator and to maintain its shape. Additionally, the silicone solution is poured such that it fills the entire mold and is cured under the same condition.

The posterior finger part is fabricated using a 3D printed mold for the chamber-based structure (Figure 1C) (Ilievski et al., 2011; Deimel and Brock, 2016; Galloway et al., 2016). The mold is designed using a 3D CAD program (SOLIDWORKS 2015, Dassault Systèmes Corp.) and manufactured by a 3D printer (ProJet HD3500 Plus, 3D Systems, Inc.). The build and support materials used in the 3D printer include VisiJet M3 Crystal and VisiJet S300, respectively. Afterward, silicone solution is poured into the 3D printed mold and cured at room temperature for 4 h. After curing, both silicone parts are combined with a silicone bond (Sil-Poxy, Smooth-On, Inc.) to prevent air leakage and maintain flexibility. The total length (*L*) of the soft pneumatic actuator is 70 mm.

The origami pump consists of Kapton tape (thickness 60 μm) and polypropylene (PP) film (thickness 200 μm). To make the folded cylindrical shape, we select the Kresling pattern (Kobayashi et al., 1998; Mahadevan and Rica, 2005; Kresling, 2008; Jianguo et al., 2015), which can be folded in the form of a cylinder shape from patterned triangles as an origami. The PP film is patterned by the laser cutter and utilized as a substrate of the origami structure. Each piece of the 2D patterned PP film is attached between two Kapton tapes and rolled into a cylindrical shape, as shown in Figure 1D. The original height (*H*) of the origami pump is 40 mm.

The air channel component is inserted between the soft pneumatic actuator and origami pump. The air channel component consists of a 3D printed part and an acrylic channel. Additionally, it is connected with the tendon attachment for the tendon-driven operation of the origami pump. The tendon attachment is a component that allows the pump to compress upwards through motor operation.

**Figure 2:**
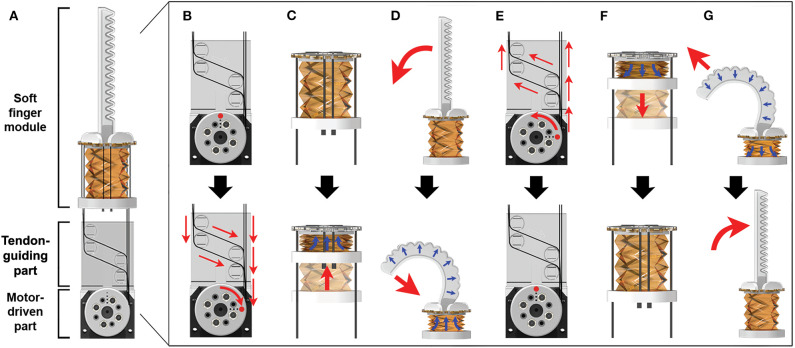
Operation mechanism of a soft finger module with the tendon-driven process. **(A)** Finger module with the motor. **(B)** Tendon-driven process with clockwise rotation of the motor. **(C)** Pump compression corresponding to tendon-driven process. **(D)** Actuator expansion procedure via air injection from pump compression. **(E)** Tendon-release process with counterclockwise rotation of the motor. **(F)** Pump release via the elastic characteristic of the origami pump. **(G)** Actuator release procedure via air extraction of the origami pump.

Finally, we add a 3D printed bottom part under the pump that is attached to seal and connect the tendons for pump operation. The bottom part is attached with the silicone bond to avoid air leakage. After the fabrication process for the soft finger module, four tendons (0.6 mm 304 grade S/S wire Rope, COSMO WIRE Co.,Ltd) are installed to the air channel, bottom of pump, and a motor (MX-28AT, ROBOTIS Co.,Ltd). To guide the tendon path, we insert the motor-driven part and tendon-guiding part, as shown in Figure 2A. The motor-driven part is manufactured by a 3D printer and connected to the motor. In addition, the tendon-guiding part is positioned between the pump and motor to change the direction of the tendons (Figure 2A). It is also utilized for the balancing operation of the origami pump by the motor.

### 2.2. Operation Principle

The operating mechanisms are classified into finger bending and finger release. The finger bending procedure is divided into three steps: (i) tendon-driven process, (ii) pump squeeze, and (iii) pneumatic actuator expansion. In the first step, the tendons are pulled by the motor using a rotating motion (Figure 2B). The second step involves compressing the origami pump. The ends of the tendons are tied to the bottom part under the soft origami pump, and they squeeze the pump by winding the tendon with the motor. Simultaneously, the air in the origami pump moves into the pneumatic actuator (Figure 2C). The actuation step is shown in Figure 2D. Specifically, when the air goes to the soft pneumatic actuator, it pushes the finger structure (Ilievski et al., 2011; Deimel and Brock, 2016; Galloway et al., 2016). The posterior finger part is designed such that it is stretched when the volume of the soft pneumatic actuator is expanded. When the posterior finger part swells, the bending angle of the actuator increases.

Conversely, the finger release procedure begins with the tendon-release process. When the motor is driven to the original position, the tension on the tendon is reduced (Figure 2E). At this time, the origami pump is recovered by the structural flexibility. This decreases the internal pressure in the soft pneumatic actuator by moving air from the actuator to origami pump (Figure 2F). Simultaneously, the chamber of the soft pneumatic actuator is reduced, and it ultimately returns to the initial position (Figure 2G).

### 2.3. Experimental Setup

**Figure 3:**
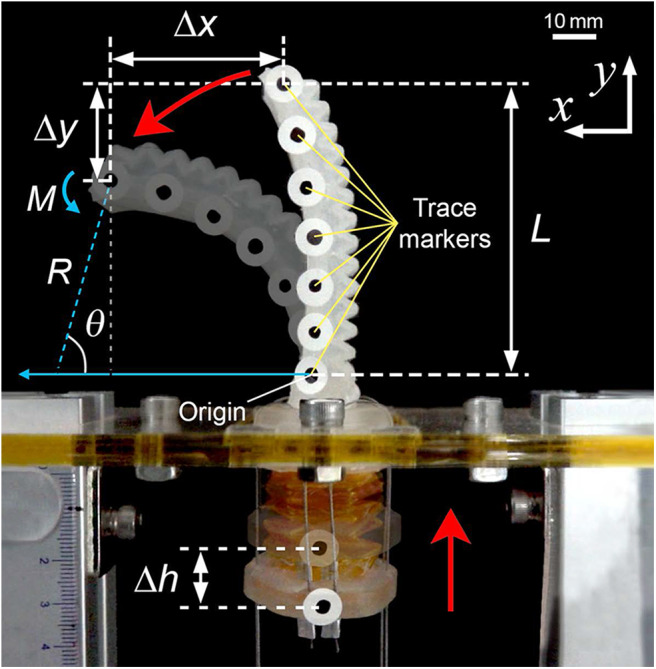
Photograph of soft finger module pump with parameters.

**Figure 4:**
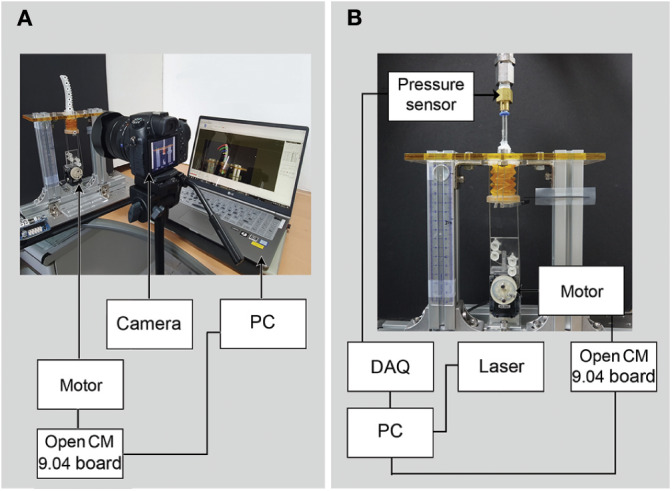
Experimental setup to measure of **(A)** optical data from the finger module operation and **(B)** output pressure from the origami pump.

We constructed several experimental setups to evaluate the performance of the soft pneumatic actuator with the tendon-driven origami pump. To measure the deformation of the soft pneumatic actuator and the height variation in the origami pump, we attach markers to the finger module (Figure 3) and record the actuation video using a camera (DSC-RX10M3, Sony Corp.) at a rate of 30 fps. Additionally, we use a post-processing tracking program (ProAnalyst Motion Analysis Software, Xcitex, Inc.) to extract the deformation data from the video. The experimental setup for tracking the deformation is shown in Figure 4A.

In addition, the blocking force was measured in full operation with soft finger module, a load cell (GS0 - 500, Transducer Techniques, Inc.) is installed on the tip of the pneumatic actuator with the contact bar. We measure the blocking force in the maximum displacement of the tendon-driven origami pump from the motor operation.

Moreover, a pressure sensor (TST-10, Nuritech, Inc.) and laser sensor (IL-100, KEYENCE Corp.) are utilized to estimate the pressure output in conjunction with the origami pump operation. A data acquisition board (USB-9162, National Instrument Corp.) is utilized to measure the outputs from the sensors. The experimental setup to observe the pressure from the origami pump is shown in Figure 4B.

In each setups, the motor is operated by the microcontroller (Open CM 9.04, ROBOTIS Co.,Ltd) and internal code to push and pull tendons.

## 3. Results

### 3.1. Finger Operation

**Figure 5:**
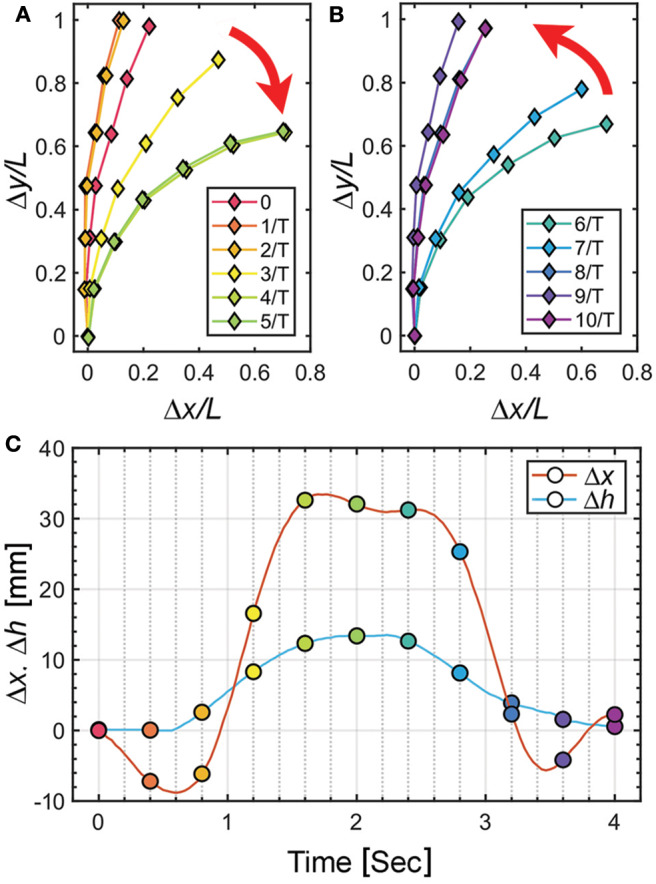
Displacement characteristics of pneumatic actuator at *f*= 0.25 Hz and Δĥ = 13.2 mm. **(A)** Normalized Δ*x* and Δ*y* in compression motion of origami pump. **(B)** Normalized Δ*x* and Δ*y* in release motion of origami pump. **(C)** Δ*h* of origami pump and Δ*x* of actuator for one cycle operation.

To characterize the deformation of the soft pneumatic actuator, we analyze the tracking results of the trace markers on the actuator. The experiment is conducted with a frequency of 0.25 Hz and the origami pump height variation (Δĥ) of 13.2 mm with the origami pump. Figures 5A,B display the deformation for the bending and release of the pneumatic actuator by sine input of the tendon-driven origami pump operation, respectively. In addition, Figure 5C shows the full displacements of the tip and the height variation Δ*h* of the origami pump to compare it with the input for a period. The positions of the markers are presented with a time step of 1/*T* = 0.4 s in Figures 5A,B. Thus, Δ*x* and Δ*y* denote the displacement variations in the *x*- and *y*-directions, respectively, at the tip of the pneumatic actuator. Additionally, we normalize Δ*x* and Δ*y* with the length of the actuator *L*.

In Figure 5C, we observe Δ*h* is close to the sinusoidal waveform, while the waveform of Δ*x* presents the distorted wave. In particular, we observe that the initial and peak regions exhibit other harmonic terms. This indicates that the observation is due to the beam oscillation. Additionally, we can compare 0 ~ 2 s and 2 ~ 4 s with bending and releasing sections corresponding to Figures 5A,B, respectively, through Δ*h*. Within, we observe a large change in periods corresponding to 2 / *T* ~ 4 / *T* and 6 / *T* ~ 8 / *T*. Furthermore, the results of Figures 5A,B indicate that the deflection is similar to the fundamental mode beam shape (Meirovitch and Parker, 2001).

### 3.2. Height and Pressure Relation

When compared to other pneumatic actuators using pressure or vacuum pumps (Jittungboonya and Maneewarn, 2019; Zhu et al., 2019), the amount of working airflow for the pneumatic actuator is limited by the tendon-driven origami pump. The working airflow is related to the volume change of the origami pump, meaning it depends on the Δ*h* of the pump.

**Figure 6:**
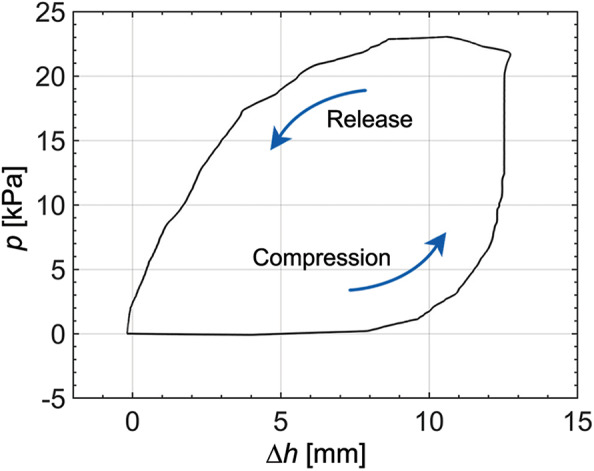
Pressure output relative to height change of origami pump at *f*= 0.25 Hz and Δĥ= 13.2 mm.

Figure 6 shows the demonstration result, and we observe the hysteresis between the pressure output (*p*) and Δ*h*. The experiment is performed at the frequency of 0.25 Hz and the height variation of 13.2 mm. When the height of the pump is increased to 8 mm from the original position, the pressure does not increase. The pressure increases gradually in the range of 8–12 mm, and increases significantly over 12 mm in compression. When the height input decreases, the pressure also gradually decreases. In the large hysteresis, we note that experiments are conducted only with the tendon-driven origami pump without a soft pneumatic actuator. The hysteresis can be attributed to the effects of the fluid dynamics in the origami pump and tendon-driven operation. In addition, the elasticity of the soft origami structure can be also one reason for the hysteresis (Chen et al., 2020).

### 3.3. Frequency Response

Another important point in the performance of the pneumatic actuator corresponds to the effect of input actuation frequency from the tendon-driven origami pump. We conducted an experiment using the pneumatic actuator at various frequency inputs (Supplementary Video 1).

**Figure 7:**
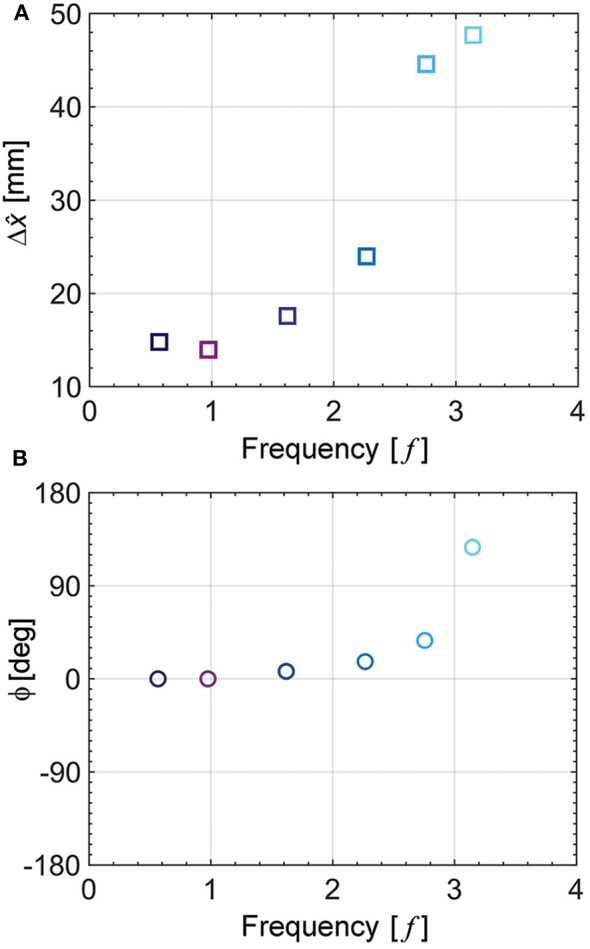
Frequency response of the pneumatic actuator at Δĥ = 4.2mm. **(A)** Maximum displacement at each input frequency. **(B)** Phase difference at each input frequency.

Figure 7 presents the result for the various input frequencies corresponding to 0.6, 1.0, 1.6, 2.3, 2.8, and 3.1 Hz at the same height variation of Δĥ= 4.2 mm. It is noted that the motor exhibits an operational limitation at higher frequencies exceeding 3.1 Hz in our test setup. Figure 7 shows the maximum displacement of the *x*-axis displacement ($\Delta \hat{x}$) and phase difference (ϕ) with the motor input. Each displacement increases slightly when the input frequency increases, as shown in Figure 7A. Interestingly, in the vicinity of 3 Hz, it exhibits significantly increased displacement. Additionally, we show the phase difference between the displacement of the pneumatic actuator and height variation in the origami pump in Figure 7B. Also, the phase difference significantly increases when it is closer to 3 Hz. The resonant frequency can be estimated when the height variation (Δĥ) of the origami pump and the *x*-axis displacement of the fingertip $\left(\Delta \hat{x}\right)$ have the phase difference of 90° (Cao et al., 2019). Moreover, we measure the natural frequency (*f*_*n*_) to validate the frequency response trend using the free vibration test from step displacement input (Alici et al., 2008; Aureli et al., 2009). Specifically, tweezers are utilized to provide input displacement, and a camera is used record at a high-speed mode of 120 fps. We measure the natural frequency corresponding to *f*_*n*_ = 2.95 Hz from the free vibration test. The value of the natural frequency corresponds to the result shown in Figure 7.

### 3.4. Blocking Force

The blocking force represents the amount of force provided by the pneumatic actuator in a stationary condition (Spinks et al., 2005). This makes it possible to infer the operating characteristics of the actuator and to estimate the weight of the object that the gripper can handle. We obtain the blocking force of 0.0653 N in the full operation. Within, we compare the blocking force of the actuator with other actuators such as pneumatic actuators with an external pressure pump (Alici et al., 2018), electrohydraulic actuators (Park et al., 2020), and SMA (Lee et al., 2019). Therein, the soft pneumatic actuators using an external pump produce a blocking force of up to 1.6767 N with a material with an elastic modulus of 262.4 kPa and up to 0.5543 N with an elastic modulus of 48 kPa. It is inferred that the pneumatic actuators using an external pump can produce a bigger blocking force. In addition, the blocking forces of the electrohydraulic and SMA actuators are 0.08 N and 0.89 N, respectively. Typically, the blocking force is proportional to the flexural rigidity of the actuator (Alici et al., 2018).

### 3.5. Pressure and Angle Relation

We investigate the degree of curvature of the actuator bending relative to pressure from the tendon-driven origami pump. Specifically, we conduct two tests: (i) actuation as the varied height of the origami pump and (ii) pressure from the pump as the varied height. The two tests are performed for the same Δĥ which corresponds to the 3.2, 4.2, 7.4, 9.3, and 15.3 mm.

**Figure 8:**
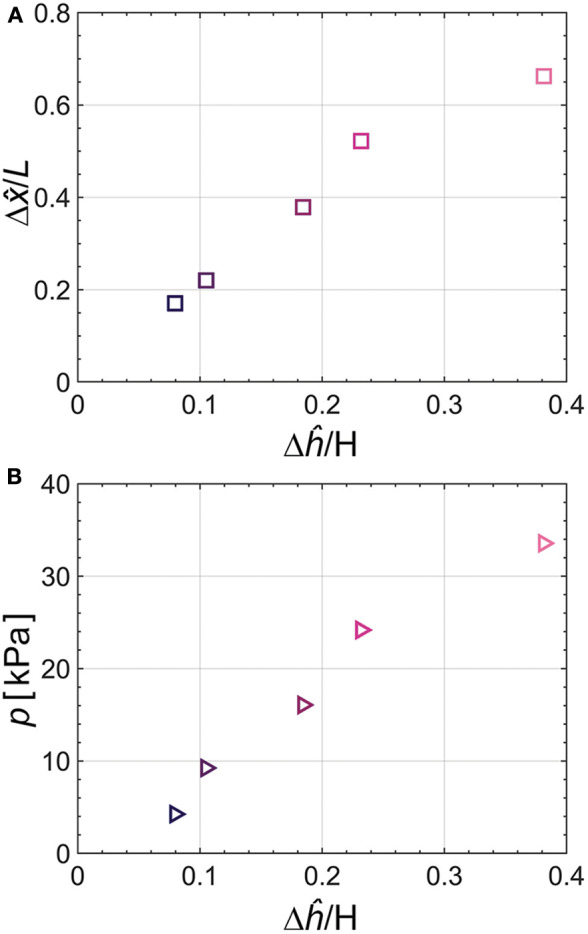
**(A)** Relation between normalized pump height and actuator displacement in *x*-direction. **(B)** Relation between normalized height and output pressure of the origami pump. Test input frequency corresponds to *f*= 1 Hz.

**Figure 9:**
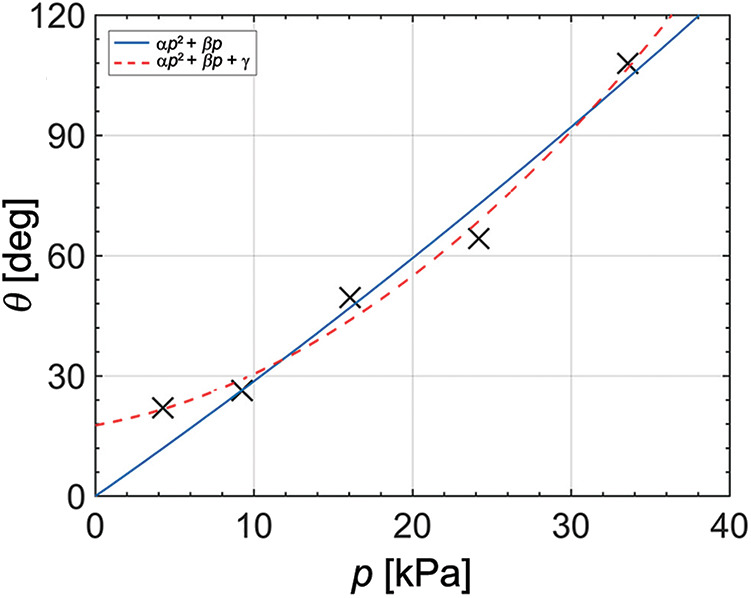
Measurement results and fitted data of actuator's bending angle and pressure from the origami pump.

Figure 8A shows the relation between normalized $\Delta \hat{x}$ and Δĥ. The result of $\Delta \hat{x}$ is normalized by *L*, and Δĥ is normalized by *H*. The output result of $\Delta \hat{x}/L$ tends to proportionally increase when input height (Δĥ/*H*) increases. Figure 8B shows the output pressure relative to the normalized Δĥ. We also observe that the pressure from the origami pump increases when the normalized Δĥ increases. Thus, we combine each result of $\Delta \hat{x}/L$ and *p*. Specifically, we evaluate the relation between the actuator's bending angle (θ) and *p*. The bending angle of θ is obtained as follows (Alici et al., 2018):

(1) $$\begin{array}{r} \begin{array}{c} \Delta \hat{x}=R\left(1-cos\theta \right)\\ L-\Delta ŷ=Rsin\theta \end{array} \end{array}$$

where *R* denotes the radius of the curvature of the bending angle as shown in Figure 3. Figure 9 and Table 1 show the bending angle at each input pressure. The bending angle of the pneumatic actuator relative to pressure was reported in (Alici et al., 2018) as follows:

(2) $$\begin{array}{r} \theta \left(p\right)=\alpha p^{2}+\beta p \end{array}$$

where $\alpha =\frac{LA^{2}e}{A_{w}E^{2}I}$ and $\beta =\frac{LAe}{EI}$, *A* denotes the inner surface area of the chamber, *e* denotes the offset between the center of pressure and the neutral axis, *A*_*w*_ denotes cross-section area of outside, *E* denotes the effective elastic modulus of the actuator, and *I* denotes the moment of inertia of the actuator. By fitting our result into Equation (2), we obtain θ(*p*) = 1.763 × 10^−10^*p*^2^+4.829 × 10^−5^*p* [rad] with an R-square value corresponding to 0.9595. The blue solid line in Figure 9 represents the fitting result. Furthermore, we can estimate the elastic modulus by using the coefficients of the fitted equation. In particular, by dividing α by β, we obtain the following relation

(3) $$\begin{array}{r} \frac{\alpha }{\beta }=\frac{A}{A_{w}E} \end{array}$$

where *A* and *A*_*w*_ are obtained from the 3D CAD program, and the values are 2.07 × 10^−4^ mm^2^ and 5.72 × 10^−5^ mm^2^, respectively. In this case, the elastic modulus is calculated as 990 kPa. The calculated value is relatively larger than the elastic modulus of the actuator material, which is known as 30-43 kPa (Larson et al., 2016; Yang et al., 2016; Jang et al., 2017). Also, we observe that the first test point does not match the fitted line. We assume that the actuator exhibits an initial curve, and we attempt to modify the relation as follows:

(4) $$\begin{array}{r} \theta \left(p\right)=\alpha p^{2}+\beta p+\gamma \end{array}$$

where γ denotes the constant term by the initial curve of the pneumatic actuator. The red dashed line in Figure 9 shows the fitted data given the initial curve. The fitting result corresponds to θ(*p*) = 1.017 × 10^−9^*p*^2^ + 1.217 × 10^−5^*p* + 0.3089 [rad] with R-square value of 0.9877. We estimate that the actuator exhibits the initial curve corresponding to 17 degrees. Additionally, we use the relation between α and β and obtain *E* = 43.28 kPa, which is compatible with that in previous studies (Larson et al., 2016; Yang et al., 2016; Jang et al., 2017).

**Table 1:** Bending angle of pneumatic actuator at different pressure values.

***p* [kPa]**	**θ [deg]**
4.25	22.08
9.25	26.38
16.67	49.52
24.18	64.25
33.56	107.95

Moreover, the natural frequency of the pneumatic actuator is as follows (Meirovitch and Parker, 2001):

(5) $$\begin{array}{r} f_{n}=\frac{3.5160}{2\pi }\sqrt{\frac{EI}{m_{l}L^{4}}} \end{array}$$

where *m*_*l*_ = 0.11 kg/m is mass per unit length. By applying the elastic modulus of 43.28 kPa, the moment of inertia is estimated as 1.80 × 10^−9^ kg· m^2^.

### 3.6. Soft Gripper

**Figure 10:**
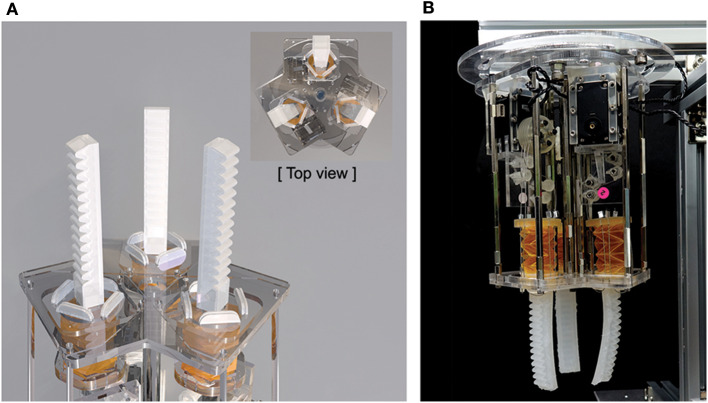
**(A)** Design of soft gripper with three soft finger modules. **(B)** Photograph of full soft gripper system.

We designed a soft gripper using the three proposed finger modules with each motor. Each module is positioned at each angular point of the triangle, as shown in Figure 10A. The body of the gripper consists of an acrylic plate, which is manufactured with a laser cutter. The completed soft pneumatic gripper system with tendon-driven origami pump is shown in Figure 10B, and the grasping motion of the gripper is shown in Supplementary Video 2.

**Figure 11:**
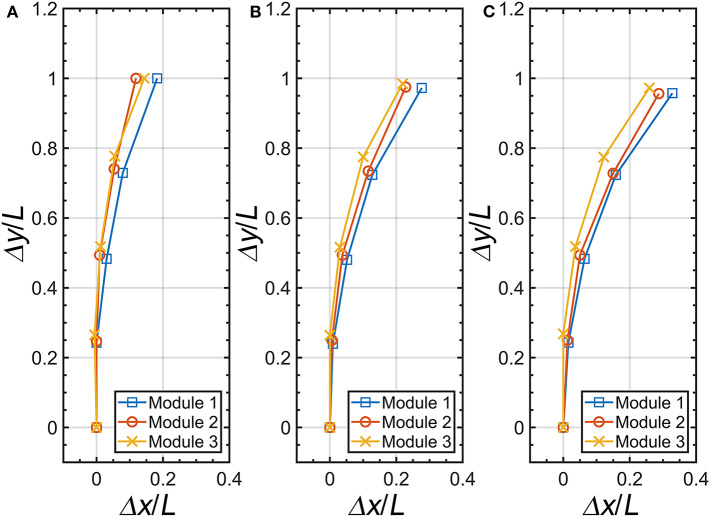
Position of the pneumatic actuator relative to the height variation of the origami pump. **(A)** Δĥ/*H* = 0, **(B)** Δĥ/*H* = 0.13, and **(C)** Δĥ/*H* = 0.19.

Within, we measured the displacements of the three finger modules with the experimental setup shown in Figure 4A. Figure 11A describes the initial position of each pneumatic actuator module, and Figures 11B,C present the maximum displacements at Δĥ/*H* = 0.13 and 0.19, respectively. The trend of initial position and maximum displacement of each pneumatic actuator are morphologically similar at same inputs. However, we can observe the deviation of each displacement caused by the handmade fabrication procedure. This results in a height difference of 2.5 mm between the tip of the actuators during the operation.

**Figure 12:**
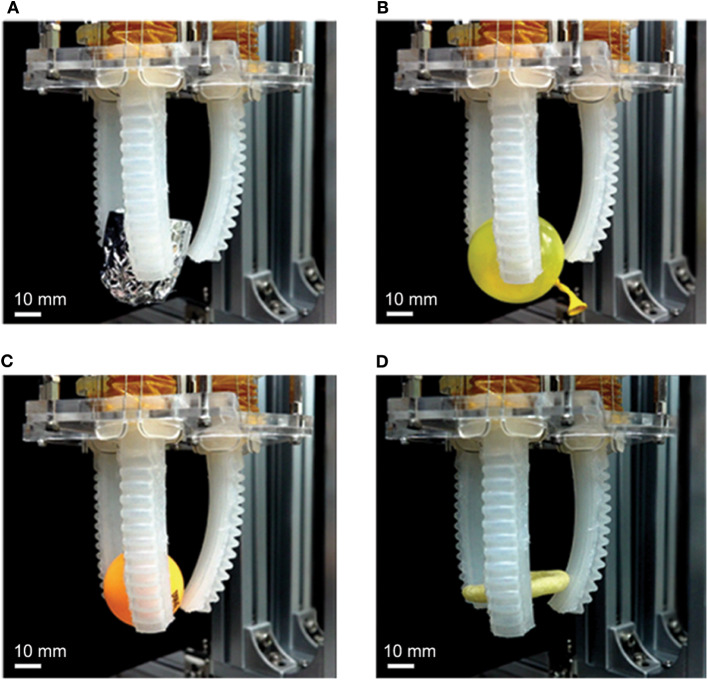
Grasping performance of soft gripper with **(A)** an aluminum cup, **(B)** a balloon **(C)** a table tennis ball, and **(D)** a ring shaped snack.

Moreover, we conducted several grasping experiments with diverse materials and shapes. The capability of the soft gripper performance is shown in Figure 12. Specifically, the soft gripper grasps an aluminum cup, balloon, table tennis ball, and snack (Supplementary Video 3). The results indicate that the soft gripper exhibits novelty in terms of grasping brittle and crumbly objects. Additionally, the demonstration shows the feasibility of the pneumatic gripper without external air compressor.

## 4. Conclusions

In this study, we designed a soft pneumatic actuator with a tendon-driven soft origami pump. We conducted a series of experiments and analyses to evaluate the performance of the actuator. Specifically, we examined the movement characteristics, frequency response, blocking force, and pressure and bending angle relation. In addition, we demonstrated the grasping of the soft gripper consisting of three pneumatic actuators.

The results indicated that the operation of the pneumatic actuator exhibits the hysteresis characteristics. We suspected that the characteristic is caused by the fluid dynamic effect of the origami pump, elasticity of the origami structure, tendon-driven operation, and soft pneumatic actuator. Additionally, the results of the frequency response experiment suggested that the natural frequency of the pneumatic actuator is approximately 3 Hz. Furthermore, we derived the relation between the bending angle of the pneumatic actuator and pressure from the origami pump. The bending angle corresponded the second order polynomial function of the pressure. Also, we calculated effective elastic modulus of the actuator from the experimental data, and the value was comparable to that in extant studies.

From the practical and methodological perspectives, the main contributions of our studies are as follows: 1) addressing the feasibility of a soft origami pump using the tendon-driven principle with a pneumatic actuator, and 2) demonstrating a soft pneumatic gripper module that can be utilized as a robot manipulator without external air supplying equipment.

## Data Availability Statement

All datasets generated for this study are included in the article/Supplementary Material.

## Author Contributions

YK and YC planned and designed the experiments. YK performed the fabrication and experiment. YK and YC analyzed the data and wrote the manuscript.

## Conflict of Interest

The authors declare that the research was conducted in the absence of any commercial or financial relationships that could be construed as a potential conflict of interest.
