# Supplementary material for: Soft Pneumatic Gripper With a Tendon-Driven Soft Origami Pump
Source: Front Bioeng Biotechnol. 2020 May 13;8:461. doi: 10.3389/fbioe.2020.00461 (PMC7274494; doi:10.3389/fbioe.2020.00461)
Supplement: Supplementary file 4 [file Data_Sheet_1.PDF]

Supplementary Material

1 SUPPLEMENTARY VIDEOS

1.1 Actuation behavior

The movie shows the feature of the pneumatic actuator under various input frequencies and different height variations of the origami pump. We conduct the experiment with various input frequencies of 0.6, 1.0, 1.6, 2.3, and 3.1 Hz at the same height variation of Δh = 4.2 mm and with the different height variations of Δh = 3.2, 4.2, 7.4, 9.3, and 15.3 mm at the same input frequency of 1.0 Hz.

1.2 Grasping motion

The movie shows the grasping motion of the gripper. The gripper is installed in the vertical position, and operated with a full operation range of the origami pump.

1.3 Grasping performance

We assess the gasping performance of the soft gripper with an aluminum cup, a balloon, a table tennis ball, and a ring shaped snack which have the characteristics of small, deformable and soft. We test the grasping performance in the vertical positions.
